# Exploring the lived experiences of mothers of disabled children with profound and multiple intellectual disabilities in Saudi Arabia: understanding caregiver empowerment

**DOI:** 10.3389/fpsyg.2026.1626005

**Published:** 2026-04-14

**Authors:** Kholood Mohammed Aljaser

**Affiliations:** Special Education Department, College of Education, King Saud University, Riyadh, Saudi Arabia

**Keywords:** contemporary models of disability, disability studies, contemporary special education, parental empowerment, profound intellectual and multiple disabilities, traditional models of disability

## Abstract

**Introduction:**

Caring for profound and multiple intellectual disabilities (PIMDs) children presents greater challenges than caring for mild-to-moderate disabilities children. Therefore, the life experiences of mothers, frequently the primary caregivers of such children, constitute a rich source of practical knowledge. This study aimed to examine mothers’ perception of empowerment in the context of caring for their children, aiming to enhance the contemporary understanding of empowerment within disability studies through an interpretive perspective based on lived experiences.

**Methods:**

A qualitative exploratory design was used, based on semi-structured interviews with 18 mothers of PIMDs children.

**Result:**

The results revealed diversity in mothers’ perceptions of empowerment, with each mother’s understanding linked to her personal context and experience. However, common patterns emerged reflecting similar childcare experiences. The findings were organized into five main dimensions representing the areas of empowerment perceived by mothers.

**Discussion:**

The discussion suggests that maternal empowerment is not understood as a static state or a direct result of institutional support but rather as a dynamic process gradually shaped by lived experiences and daily situations. This model is described as the concept of Lived-Experience-Based Empowerment (LEBE). A comparison with prevailing disability models indicates that, although this concept shares some assumptions, it expands upon them by highlighting the pivotal role of lived experience in shaping the meaning of empowerment.

## 1 Introduction

The concept of empowerment is central to disability studies and is linked to theoretical shifts that have transformed the understanding of disability from traditional models, which view it as an individual problem attributed to impairment, to contemporary models of disability that see it as a phenomenon shaped within broader social and structural contexts (Dan, 2020). Academic and professional interest in empowerment has grown, as it has emerged as a central value in the fields of social work, rehabilitation, community psychology, and policymaking. This increased interest reflects growing criticism of the medical model’s limitation, increasing awareness of the importance of involving disabled people and their families in decision-making, and accumulating evidence indicating that empowerment is linked to improved social functioning, enhanced quality of life, and greater social inclusion (Dan, 2020; Stoykova, 2021; Tören and Aslan Açan, 2024). This shift has contributed to a re-evaluation of the roles of families, particularly parents of disabled children, as key actors in supporting their children’s development, rather than merely recipients of services or care, as indicated by literature highlighting that contemporary care has empowered parents of disabled children’s central aim. It aims to strengthen their ability to support their children and actively participate in decision-making, unlike traditional models that prioritize expert input only and marginalize parents (Fischer and Goodley, 2007; Federici et al., 2008; Mahmic et al., 2021).

Contextually, the literature on empowering parents of disabled children has expanded, recognizing them as pivotal partners in their children’s development. Scholars have widely reviewed literature on empowerment for parents of mild to moderate disabled children. Emphasizing that such empowerment enhances parents’ ability to manage daily challenges, improves access to resources, reduces stress (Nachshen and Minnes, 2005), strengthens cooperation with service providers, increases parents’ influence over decisions and services, and encourages active participation in designing programs that meet their children’s needs (Alsem et al., 2017; Banach et al., 2010). This review included studies from the United States (Banach et al., 2010; Burke et al., 2019, 2021; Koren et al., 1992; Murray et al., 2013), Israel (Itzhaky and Schwartz, 2001), the Netherlands (Alsem et al., 2017), Australia (Dempsey and Dunst, 2004), South Korea (Han et al., 2018), Canada (Nachshen and Minnes, 2005; Weiss et al., 2012), and Japan (Wakimizu et al., 2011, 2017, 2022). Similar research has been conducted in the Arabic context, including Egyptian (Asran, 2018; Alnemr, 2020; Halat, 2012) and Jordanian studies (Alahmad, 2017; Almosaadah, 2010; Damra, 2015), as well as in Saudi Arabia (SA), although the published studies are limited. For example, Aldalbahi (2018) conducted a study in Dawadmi City examining social support and psychological empowerment among working and nonworking mothers of mild intellectual disabled children.

Despite this widespread interest and notions of empowerment developed within the context of mild to moderate disabilities, concerns have been raised regarding their conceptual applicability to the highly specific contexts of severe disabilities such as PIMDs. This classification represents one of the most severe and complex forms of chronic disability, as it affects multiple organ systems and requires lifelong specialized medical and educational care (Rousseau et al., 2023). As conceptualized by Nakken and Vlaskamp (2007), this condition is characterized by two core features: profound intellectual disability and profound motor disability, along with additional severe impairments, including visual and hearing limitations, chronic health problems, and minimal verbal understanding or engagement. Communication is also perceived as problematic because of the severity of disabilities, which causes children to use nonverbal and non-symbolic expressions through gestures, facial expressions, sounds, and body movements that are not easy to understand for those not close to the child (Aljaser, 2017). As Rankin and Regan (2004) argued, the condition is marked by both breadth, resulting from multiple co-occurring disabilities, and depth, owing to the severity of these impairments.

This approach characterizes caregiving as highly intensive, featuring disordered and unsettled parenting experiences (Aim et al., 2023; Tadema and Vlaskamp, 2010; Woodgate et al., 2015). Parents engage in hands-on care tasks such as feeding, bathing, lifting, positioning, and operating medical equipment (Geuze and Goossensen, 2021; Luijkx et al., 2017); coordinate complex medical appointments and services (Geuze and Goossensen, 2021); marshal resources including transportation and financial aid (Aim et al., 2023; Woodgate et al., 2015); and remain alert to unpredictable crises such as seizures and respiratory distress (Geuze and Goossensen, 2021), which become increasingly concerning as their children grow older (Luitwieler et al., 2021). Empirical evidence reveals that such parents dedicate approximately 4–6 h to direct care daily and an additional 6.5 h to uninterrupted supervision, with mothers bearing most of the burden (McCann et al., 2012). Consequently, parents of severe disabled children a lower quality of life than parents of mild-to-moderate disabled children (Luitwieler et al., 2021; Sulaimani et al., 2023). This experience includes physical strain from caregiving responsibilities, sleep deprivation that compromises their own health, financial difficulties (Baumstarck et al., 2025; Geuze and Goossensen, 2021), emotional challenges stemming from chronic anxiety about their child’s survival, persistent fear of premature death, relentless cycles of grief (Baumstarck et al., 2025; Geuze and Goossensen, 2021; Smith et al., 2015), and social isolation due to the inability to leave their child unattended as well as stigma from extended family members (Huang et al., 2010; Woodgate et al., 2015).

Therefore, revisiting the concept of empowerment in these contexts is a cognitive necessity for understanding how it is shaped and what it means in the context of intensive, long-term caregiving experiences. However, research on empowering parents of PIMDs children remains limited and largely quantitative, relying on standardized tools, such as parental empowerment scales (Family Empowerment Scale, FES; Koren et al., 1992) and quantitative work in Norway (Kalleson et al., 2019) and Japan (Fujioka et al., 2015; Wakimizu et al., 2016). Such work creates a gap in understanding empowerment because standardized scales, even when they have high psychometric validity, may overlook important aspects of parents’ lived experiences and limit the identification of empowerment elements shaped by different contextual challenges (Singh et al., 1995). For instance, based on its items, FES (Fujioka et al., 2015; Kalleson et al., 2019) conceptualizes empowerment as a parent’s ability to navigate and participate in existing service structures without fully addressing the wider systemic issues and broader social change. To mitigate these challenges, scholars often use qualitative designs that capture experiences that cannot be measured quantitatively (Tenny et al., 2022). However, the qualitative work on empowerment in PIMDs research is limited. Among the few published works is a study by Reeder and Morris (2021) in the UK focusing on how 14 parents of long-term disabled children experienced empowerment during their interactions with pediatric health professionals. Although relevant, the study failed to explicitly explain the nature of children’s disabilities. Therefore, some parents may have been caring for children who did not meet the criteria for PIMDs. The study also provides only micro-level insights from specialized healthcare settings, without considering the wider challenges that parents face in daily life.

Therefore, to expand the understanding of empowerment beyond the narrow frameworks of functional needs assessment and health contexts, understanding how empowerment is embodied in the daily lives of caregivers is essential as they care for their children and interact continuously with the environment and services (Dempsey and Dunst, 2004). The lived and accumulated experiences of parents form the basis for a deeper understanding of empowerment, as daily care practices and continuous interaction with different systems yield knowledge derived from direct encounters with the challenges of disability and continuous adaptation to its requirements (Clifton et al., 2025; Gona et al., 2018).

This topic gains added significance in the SA context, where services for PIMDs children are distributed among multiple entities and mothers often bear the primary responsibility for daily care. Given the rapid social and institutional transformations in SA, particularly in light of the Saudi Vision 2030 objectives related to the quality of life and enhanced community participation, examining how the concept of empowerment is shaped within this specific social and cultural framework becomes pertinent. Since he concept of empowerment is shaped by cultural and institutional contexts (Hui et al., 2004; Monje-Amor et al., 2021; Tören and Aslan Açan, 2024). which supports the need to study empowerment from within the lived experience of the targeted local populations. Given these circumstances, how mothers of PIMDs children perceive the concept of empowerment in their daily lives and how this concept is formed through their ongoing interaction with caregiving roles, service systems, and the broader social context requires deeper understanding. Therefore, this study aimed to explore mothers’ perceptions of empowerment and analyze the dimensions through which it manifests in their lived experiences. To achieve this goal, the study addressed the following questions:

- *How do mothers of PIMDs children perceive the concept of empowerment through their lived experiences?*
- *How does their concept of empowerment intersect with models of disability, and where does it go beyond them?*

## 2 Methodology

### 2.1 Study design

Given the shortcomings of quantitative studies and their reliance on standardized tools that do not reflect the lived experiences of parents of PIMDs children, adopting an approach that captures the phenomenon of empowerment through verbal description, interpretation, and discussion is essential to reveal meanings that cannot be reached through traditional quantitative methods. Therefore, this study adopted a qualitative, exploratory, and constructivist approach, which sought to explore the concept of empowerment as perceived by mothers through their life experiences and then develop an empowerment model based on field data. This approach allows for a deep understanding of the phenomenon through verbal description, interpretation, and discussion (Anderson and Arsenault, 2002; Matthews and Ross, 2010), enabling the study to capture the individual meanings that mothers of PIMDs children ascribe to the concept of empowerment and the life experiences that formulate this concept.

### 2.2 The researcher’s position and reflectivity

Given that this study adopted a qualitative interpretive approach, clarifying the researcher’s position and role in the data collection and analysis process and the potential impact this might have on interpreting the results was essential. This study stems from my academic background in special education, particularly in dealing with PIMDs, which has shaped my interest in family support and empowerment in this specific context. This heightened my sensitivity to the complexities of mothers’ experiences and helped me build a trusting relationship during data collection. Simultaneously, I was aware that my professional experience might influence interpretation; therefore, I adhered to a systematic, reflective approach that included continuous frequent revisits of the original data, documentation of analytical decisions, and ensuring that the findings were based on the participants’ own narratives of their lived experiences, rather than preconceived assumptions.

### 2.3 Data collection

Data were collected through semi-structured individual interviews that allowed participants to articulate their views on abstract topics (empowerment) (Gray, 2014). This enabled participants to express themselves freely and on their own terms, producing deeper insights and a more comprehensive understanding of their values and perspectives (Rubin and Rubin, 2012). Individual interviews also allow for the observation of nonverbal cues, which play a vital role in interpreting perspectives (Aljaser, 2020; Matthews and Ross, 2010).

The interview protocol included a set of open-ended sub-questions designed to allow mothers to freely express their conceptions of empowerment and recall life experiences and situations that contributed to shaping this concept. This was accomplished by focusing on the following areas: (1) Personal experiences and child and family characteristics, which were captured using questions such as, “Tell me about yourself and your child,” “What are your child’s needs? Do you see them as multiple?” and “What were your feelings when your child was born?” and questions about the stages of care and their difficulties compared with the current stage; (2) Understanding empowerment from the mothers’ perspective, targeted using questions such as, “How do you define empowerment?” and “What is the opposite of empowerment?,” “How will empowerment help you, and how will it help your child?” “How does this concept manifest in daily practice?” and “Why do you see this as empowerment for you?” (3) Factors influencing empowerment, captured with questions such as, “What is your source of strength in caring for your child?” “What are the obstacles to maternal empowerment?” (4) The impact of parental and school characteristics on feelings of empowerment and examples of life experiences in which they felt empowered. (5) Mothers’ knowledge of local and international empowerment systems. A pilot interview was conducted with two participants outside the main sample to ensure that the questions were consistent with the study objectives and clear to the participants. Revisions to the wording of some items were made before the interviews began.

Most interviews were conducted face-to-face to ensure direct communication for a better understanding of participants’ concepts of empowerment and life experiences. However, a few interviews were conducted via Zoom with mothers who lived outside the central region (the researcher’s city) or those who required additional interview sessions. After each interview, I reviewed the key points with the mothers and paraphrase what they had said to ensure that their meanings were accurately understood and to allow them to clarify any details when needed. This process helped enhance the credibility of the interpretations and the accuracy of the extracted meanings.

### 2.4 Participation

Given the typically small sample size used in qualitative research, a purposive sampling strategy was adopted to recruit participants who could provide information relevant to the study objectives (Cohen et al., 2010). Participants were included if they were mothers of PIMDs children over 2 years of age or adults and had experience in accessing local services. This study targeted mothers because they often bear primary caregiving responsibilities and are more involved in caregiving and advocacy (Burke et al., 2021; Smith et al., 2015; Tadema and Vlaskamp, 2010), which makes their life experiences more insightful.

Children’s characteristics were verified to ensure alignment with PIMDs characteristics; specifically, profound intellectual disability with a score below 25–20, severe motor disability, and accompanying sensory and health disabilities; mothers of children with multiple disabilities were excluded (Aljaser, 2025). In SA services for disabled children are distributed among several government ministries, each providing specific types of support. While the Ministry of Education supports mild and moderate disabilities children through inclusion programs and specialized centers, the Ministry of Human Resources and Social Development provides care for severe disabled children in residential and daycare centers. Children who do not attend these centers receive health and rehabilitation services from the Ministry of Health (Aljaser, 2025).

The participants were recruited through various channels, including special education centers, hospitals, and social media platforms. The selection process also relied on referrals from participating mothers, who helped connect the researcher with additional mothers. Participants were successfully recruited from diverse geographic areas of SA to obtain a range of caregiving experiences. This included the central region, which comprised most of the sample, along with the southern and western regions (Table 1 summarizes the characteristics of the participants).

**Table 1:** Demographic characteristics of the study participants.

Participants	Employment status	Household income level	Marital status	Educational level	Birth order of the child	Type of support provided to the child
p1	Unemployed	Middle-income	Married	Middle level education	First child in the family	Special education center for severely disabled
p2	Unemployed	Middle-income	Single mother	Middle level education	Last child in the family	Special education center for severely disabled
p3	Unemployed	Middle-income	Married	Middle level education	Last child in the family	Medical support
p4	Unemployed	Middle-income	Single mother	Middle level education	First child in the family	Special education center for severely disabled
p6	Unemployed	Middle-income	Married	Middle level education	Last child in the family	Medical support
p7	Unemployed	Middle-income	Married	Middle level education	Last child in the family	Medical support
p8	Unemployed	Middle-income	Single mother	Middle level education	First child in the family	Medical support
p9	Unemployed	Middle-income	Married	Middle level education	First child in the family	Special education center for severely disabled
p10	Unemployed	Middle-income	Married	Middle level education	Last child in the family	Medical support
p11	Unemployed	Middle-income	Married	a middle level of education	Middle child in the family	Medical support
p12	Employed	Middle-income	Married	Middle level education	Middle child in the family	Special education center for severely disabled
p13	Employed	Middle-income	Married	Middle level education	First child in the family	Medical support
p14	Employed	Middle-income	Married	High level education	Middle child in the family	Special education center for severely disabled
p15	Employed	Middle-income	Married	Middle level education	Middle child in the family	Medical support
p16	Employed	Middle-income	Married	High level	Last child in the family	Medical support
p17	Employed	Middle-income	Married	Middle level education	Middle child in the family	Special education center for severely disabled
p18	Employed	Middle-income	Married	Middle level education	Middle child in the family	Medical support

Participation in the study was encouraged through flexible scheduling, repeated invitations, and reassurance regarding the value of mothers’ perspectives. Some mothers, especially in the early stages of childcare, initially lacked time or emotional energy; however, a calm conversational style that created a safe and comfortable setting reduced their hesitation and enabled many to complete the interviews. The sample size was determined according to qualitative research principles that prioritized analytical depth over statistical generalization. Data collection continued until subject saturation was reached. The sample size was gradually determined concurrently with the data collection and analysis. As the interviews progressed, the data were examined continuously to observe the emergence of new meanings or patterns. Subsequent interviews revealed a clear repetition of the participants’ experiences and issues, with further interviews failing to yield new ideas or themes related to the study topic. Based on this analytical repetition, data collection was discontinued once a sample of 18 mothers was reached, as this number was deemed sufficient to support the qualitative analysis and achieve the study objectives.

### 2.5 Data analysis

The codes were developed manually using a thematic analysis approach (Braun and Clarke, 2006) without using any qualitative analysis software. This approach included a series of systematic steps, beginning with repeatedly reading the interview transcripts (the raw data) to form a preliminary and comprehensive understanding of their content; revisiting and listening to the recordings while reading the transcripts verbatim; and forming initial notes about possible ideas, feelings, and patterns, which helped the researcher understand the depth of the data before commencing coding. This was followed by the initial coding of relevant sentences and phrases, where recurring concepts and defining features were identified inductively based on the data, without imposing any preconceived theory (Braun and Clarke, 2006) and paving the way for the emergence of new meanings that are not captured by ready-made theory (Matthews and Ross, 2010). To answer the first question concerning mothers’ concepts of empowerment, their narratives were analyzed by linking their definitions of the concept to the life experiences they had recounted during the interviews. This connection between “what the mother says” and “what she actually experiences” indicates that empowerment comprises the intersection of personal understanding with the lived experience of mothers.

Data analysis began after a series of initial interviews during which the transcripts were repeatedly transcribed and read to ensure a thorough understanding of the content. The data were manually coded line-by-line, focusing on phrases that reflected participants’ experiences and meanings related to the study topic. As the analysis progressed, the codes were continuously reviewed and compared across interviews, with similar codes being merged, refined, and irrelevant ones eliminated. This iterative process led to the progression from initial codes to broader categories and then to overarching themes, which were subsequently grouped into larger analytical clusters to minimize overlap and enhance the level of analytical abstraction. These procedures ensured that the results were directly based on the participants’ data and reflected the common patterns in their experiences.

Finally, the results were interpreted and placed within the broader context of previous literature, enhancing their objectivity and clearly demonstrating how this study bridges existing knowledge gaps and expands our understanding of empowerment in disability studies.

### 2.6 Ethical consideration

This study was approved by the Research Ethics Committee of King Saud University and adhered to the university’s King Saud University Research Ethics Policy (2015) regarding studies involving human participants, data collection and storage, and the management of personal information. The study objectives and procedures were explained to all participants to ensure their understanding of the research and the importance of their participation. Participation was entirely voluntary, and the participants were informed of their right to decline to answer any question or withdraw at any time before or during the interview without repercussions. They were also informed that their individual interviews would be recorded to ensure accurate documentation and data analysis. To guarantee confidentiality, participants’ names were replaced with codes, and all collected data were used solely for scientific research purposes.

### 2.7 Trustworthiness

Trustworthiness was ensured through validity, reliability, reproducibility, and confirmability (Matthews and Ross, 2010). Validity was enhanced by collecting data directly from mothers, frequently reviewing audio recordings, clarifying interview questions as needed, and discussing the collected data with participants to ensure accuracy and validity. Reliability was addressed through pilot interviews, which helped refine the interview tools and techniques, as well as regular revisions to ensure consistency throughout the data collection process. Additionally, the analysis and results were reviewed by expert colleagues in the field to ensure methodological consistency and accuracy of interpretation, along with frequent references to the original texts, to verify that the topics accurately presented the participants’ concept of empowerment and the life experiences shaping it, ensuring that all important and recurring ideas were included in the final analysis. To enhance reproducibility, purposeful and representative samples were used along with detailed descriptions of the participants and their contexts, allowing readers to assess the applicability of the findings in similar settings. Confirmability was achieved by preserving the original data from the recordings and reflective notes, minimizing researcher bias, and ensuring that interpretations were based directly on the data. Participants were also given ample time to reflect during the interviews, and post-analysis communication was used to clarify answers when needed.

## 3 Results

### 3.1 Dimensions of empowerment as experienced by mothers

The analysis revealed diversity in the mothers’ perceptions of the concept of empowerment, with each mother expressing an understanding that reflected her personal context and circumstances. However, the analysis also uncovered common patterns and meanings that recurred across the interviews, indicating shared experiences that contributed to a cross-sectional understanding of empowerment among the participants. Accordingly, the results were organized into five main dimensions representing the areas of empowerment as perceived by the mothers, with each dimension branching into several related subthemes.

#### 3.1.1 Dimension one: service-oriented empowerment

This dimension reflects mothers’ perceptions of empowerment as being linked to the effectiveness of the service system and its ability to respond to the needs of their children and families. Empowerment is not measured merely by the availability of services but by the quality, speed, and consistency of the response. Through actual interactions with the support system in daily life, this understanding was crystallized across five subthemes drawn from their lived experiences.

##### 3.1.1.1 Empowerment as rapid, consistent, and continuous support

Mothers consistently associated empowerment with receiving prompt and sustained support over time. The absence of early and ongoing provision was not merely inconvenient but had lasting consequences for how mothers were able to establish stability in their caregiving role. One mother explained:

Empowerment is a parenting demand. It is just like preparing a life for my daughter and me…she was eight years old when I settled down…continuous support from her birth would have helped.

Her framing of empowerment as a “parenting demand” positions it as a fundamental entitlement rather than an optional addition to the care system. The eight-year delay, as described by the participants, illustrates how the absence of early intervention, which goes beyond an exclusive focus on medical treatment or staying at home, forces mothers to live in a state of prolonged uncertainty, making it difficult for them to develop a positive outlook on caring for their children or to build a stable daily routine. Her use of the phrase “preparing a life” implies that empowerment is not a single event but a cumulative, forward-looking process that must begin at the point of diagnosis to be meaningful. This indicates that empowerment was linked to enhancing her ability to cope with future uncertainty and striving to build a more stable life path for her children. Another mother reinforced the link between the continuity of support and empowerment:

After the teacher changed, I noticed the difference. I need ongoing support; I want my daughter to have a good and useful time. I keep receiving calls from her [the teacher] complaining about my daughter’s lack of interaction.

The mother’s remarks highlight how staff turnover negatively impacts not only her child’s progress but also her sense of empowerment. The transition from a supportive professional relationship to one characterized by complaints about the child’s behavior indicates a decline in the quality of support provided. This underscores that for mothers of PIMDs children, where developmental gains are gradual and fleeting, the instability of services poses a direct threat to the child’s well-being and the mother’s confidence in the system. In the local context, this reveals a gap between the nature of the needs of PIMDs, which require continuity of support and professional stability, and the operational practices in some educational environments (special education centers) that are accustomed to managing less complex cases, thereby rendering stability a crucial element in the quality of support provided. This is directly reflected in the mother’s sense of empowerment, as the stability of support is linked to her confidence in the system’s ability to respond to her child’s long-term needs. Both quotes expressed a lack of service stability, whether through delays or staff turnover; the speed of response and continuity of support emerged as crucial factors in their sense of empowerment.

##### 3.1.1.2 Empowerment as an integrated support system

Given the complex and multi-layered needs of PIMDs children, all mothers, either directly or indirectly, identified fragmented service delivery as a major barrier to empowerment. For these families who must interact with intervention, therapy, and medical providers simultaneously, disjointed communication between services placed a disproportionate burden on mothers to act as intermediaries. One mother explained:

Cooperation is empowering. The delay in providing comprehensive support will affect them more…duplication of work; everywhere I go, I get the same questions. Sometimes, I need to explain my child’s condition again and again.

The repetitive questioning that she described was not simply an administrative inconvenience. It forces mothers to relive the emotional weight of their child’s diagnosis at each new service point and signals a system that lacks internal communication. Her association of “cooperation” with empowerment is significant because it locates the problem not within the mother’s capacity but within the structural design of the service system itself. Another mother offered the following complementary perspective:

Empowerment is streamlined; no need to work hard to access service. I received a call to admit my daughter to the center [Special education center]…but on the condition that physical facilities would be provided for her.

Her definition of empowerment as “streamlined” reflects an expectation that the system will communicate effectively with families rather than leaving mothers to navigate bureaucratic procedures alone. Furthermore, given the limited number of specialized centers for PIMDs where the focus is often on medical support, the requirement for admission to some schools/centers to meet specific accessibility criteria, reveals an organizational structure in which access to support is determined by institutional considerations. This places responsibility for compliance with requirements on the family, rather than managing it through direct coordination between relevant parties. In the local context, where services for PIMDs children are distributed across multiple sectors with varying coordination mechanisms, empowerment is understood as the system’s ability to function harmoniously and fulfill its responsibilities, not as an individual effort by the mother to overcome procedural complexities.

##### 3.1.1.3 Empowerment as collaborative partnership

Some mothers conceptualized empowerment as being rooted in the quality of their relationships with service providers. These accounts have moved beyond satisfaction with services to describe the relational dynamics of respect, communication, and shared decision-making. One participant said:

Empowerment…[is] providing what should be provided. She [the physiotherapist] keeps me up to date with everything and asks me for information and feedback.

Her definition of empowerment as “providing what should be provided” reveals a normative conception of empowerment based on the idea of entitlement, where support is not viewed as an additional initiative but as an institutional obligation that is supposed to be fulfilled without constant demands from the family. This understanding was embodied in the physical therapist’s communication with her and his request for information about her child, reflecting her perception of empowerment as a partnership based on knowledge sharing and recognition of the mother’s expertise and integrating her role in the service delivery process. Her participation in professional dialogue reinforced her sense of being an active participant in the support process and not a passive recipient. In the local context, where some professional relationships are managed within traditional frameworks that tend to centralize the professional’s role in decision-making, this participatory approach is of particular significance. It redistributes roles between the family and the professional and grants the mother a recognized knowledge base within the rehabilitation process. Thus, empowerment is understood here as the mutual recognition of expertise and not merely the implementation of one-way professional recommendations.

The following quote clearly illustrates this contrast, as the mother describes two contrasting professional experiences in two visits seeking government financial support that reflect her understanding of an empowering versus a non-empowering relationship:

Two visits in search of financial support gave me different experiences of empowerment. Initially, it was so hard trying to explain my daughter’s need for financial support… The language used made me uncomfortable. But the second visit was so easy. I just sat on the sofa, and the social worker took my daughter. I updated the papers briefly and received the support easily and effortlessly, as a right.

This narrative reveals that empowerment is not only related to the availability of the service but also to the nature of the professional relationship, which is based on mutual recognition and communication. The first visit required the mother to justify her child’s needs by using language she found distressing, placing her in a position of supplication. By contrast, the second visit was organized around her comfort and efficiency. This mother pointed out that empowerment means obtaining what is legally due without having to fight for it. This reveals that empowerment is not achieved simply by the existence of a law stipulating a right but rather through a professional relationship that practically activates this right and facilitates access to it without placing the burden of constant demands on the family.

##### 3.1.1.4 Empowerment as effective service outcomes

For some mothers, empowerment is inseparable from observable improvements in their child’s development and functioning. The tangible results of service provision served as evidence that the system’s investments produced meaningful changes. One mother described the connection as follows:

When I look at my child who has benefited from the services, I feel happy and empowerment… I felt like someone else when I started to notice how my child developed and interacted. Before, she would constantly cry and had trouble sleeping, but after the surgery and the therapy sessions, she even started communicating and playing with her brother.

The phrase “felt like someone else” suggests that the child’s transformation triggered a parallel transformation in the mother’s identity and emotional state. Contextually, empowerment is not static; it is produced by witnessing change. She illustrates how the developmental shift from constant distress to communication and play did not only alter the child’s daily life but also reshaped family interactions, allowing siblings to engage with the child in new ways. This transformation is connected to a sense of empowerment, as evidenced by the use of the word “benefit” as a measurable outcome of service provision that reinforced her sense of empowerment.

By contrast, other mothers’ experiences of service failures produced the opposite effect. One mother expressed deep frustration:

My son goes to the center [special education center] without interacting with others. I hope no one ignores him. They speak with less severely disabled and verbal children…teachers do not respect my feelings of love and faith in my child’s ability.

This case raises the same issue of the unequal treatment of disabled children within the same service setting. As she expressed, neglect of her son was disheartening because it failed to acknowledge her belief in her child’s abilities. She saw attending the center as an opportunity to receive tangible support that would make a real difference, not just a token presence. This narrative is particularly significant because it appears within the context of her definition of empowerment, indicating that, in her view, empowerment is linked to seeing a tangible and equitable benefit from the services provided. Empowerment here is not merely about providing the service but about achieving equitable outcomes that reflect the effectiveness and quality of the support, affirming that a child’s condition—regardless of severity—does not diminish their entitlement to effective service.

##### 3.1.1.5 Empowerment as conscious home-based care

Mothers also reinforced the concept of empowerment by emphasizing the importance of providing specialized care within the home, given the children’s fragile health and the difficulty of transporting them to service centers. In the local context, where some of these children do not attend school, and their support is limited to sporadic health and rehabilitation services, home visits also emerged as a key mechanism for implementing empowerment, contributing to the standardization and diversification of services within the child’s daily environment and promoting their integration. One mother explained:

Empowerment is essential for family balance through periodic and productive home visits and consultations by professionals such as social workers, occupational therapists, and others…all in the comfort of our home. My child spends most of his time in the house, especially when sick or after surgery. His sisters are also involved, which reflects positively on all of us… Changing our routine like this empowers me.

Her reference to “family balance” is analytically important because it positions empowerment not as an individual maternal outcome but as a whole-family process. The involvement of siblings in home-based professional visits creates shared learning opportunities and distributes the caregiving experience across the family unit rather than concentrating solely on the mother. Her description of the home as a space of “comfort” is not merely about physical convenience; it refers to an environment where the child is most stable and the family is most cohesive. Another mother added:

Empowerment for me involves flexible access to services by visiting me and providing me with consultations… The flexibility serves as compensation for the hard days we have… My daughter is a technology-dependent child who has outgrown the old chair. A traditional chair was provided for her, but unfortunately, her motor interaction decreased because the chair was unsuitable for her size. Wheelchairs, splints, and other equipment must be adjusted continuously and quickly within the home to maintain normal living.

Her description of flexibility as “compensation for the hard days” reveals how home-based service delivery functions as a form of systemic acknowledgement of the extraordinary burden these mothers carry. The example of an unsuitable wheelchair is of particular interest. It demonstrates how a single piece of mismatched equipment can lead to functional regression and how the pace of service response either supports or undermines a child’s participation in daily family life. Her phrase “maintain normal living” encapsulates a core aspiration shared by many mothers in this study: that empowerment means enabling their child to participate in everyday routines with comfort and dignity.

#### 3.1.2 Dimension two: cognitive and capacity-building empowerment

This dimension reflects mothers’ understanding of empowerment as an ongoing process of acquiring the knowledge and practical skills that enable them to care for their children consciously and confidently. Here, empowerment manifests as developing expertise, refining caregiving competencies, and the ability to apply specialized knowledge in daily life. For mothers of PIMDs children, whose medical needs are complex and constantly evolving, the ability to understand their condition and respond effectively to emergencies is a crucial element of their empowerment experience. In the SA context, in which structured training pathways for families, particularly for severe disabled children, are not always available, a mother’s experience is gradually shaped through direct interaction with the child and service system. This makes empowerment as much about accumulating knowledge through daily experience as it is about formal professional guidance. This understanding is explored through two subthemes.

##### 3.1.2.1 Empowerment as a parenting competency

All the interviewed mothers expressed concerns about their ability to meet their child’s needs. Empowerment, in their accounts, was closely tied to developing a positive self-perception as a capable parent, shaped by their accumulated efforts and their child’s achievements. Several mothers expressed this connection:

I learned many ways to make her happy, which was reflected in me. I feel motherly… Empowerment is a main ingredient of effective living.

I do stretching exercises daily and play games with my daughter. This makes me self-sufficient…I have learned many ways to make her happy.

When I wake up in the morning and feel that I have done something beautiful for my son, this is empowerment… My son brought out the best version of me. I understood the true feeling of happiness in any simple development. His laughter is worth the world.

These statements reveal that parenting self-efficacy is the primary driver of empowerment for these mothers. The first mother’s description of feeling “motherly” only after learning specific caregiving techniques suggests that the identity of “mother” was actively constructed through the acquisition of competence. The second mother’s reference to “self-sufficiency,” gained through daily physical exercise with her daughter, links empowerment to practical autonomy in care. The third mother’s account is particularly significant because it reframes the caregiving experience as a source of meaning and personal growth rather than a burden. Her ability to find joy in “simple development” and to describe her son’s laughter as “worth the world” indicates a cognitive reorientation in which even small milestones carry immense emotional weight. Together, these accounts position parenting competency not as a technical skill alone, but as an existential resource that sustains mothers through the daily demands of PIMD care.

##### 3.1.2.2 Empowerment as access to specialized knowledge and expertise

Mothers with PIMDs children, particularly in the early postpartum period, frequently described their struggle to cope with the rapid accumulation of diagnoses, medical terminology, and specialist appointments. As the number of professionals increased, confusion and anxiety often intensified, and in some cases, emotional withdrawal from the child occurred. Contextually, access to knowledge became the primary pathway to empowerment. One mother stated:

Knowledge, knowledge, and knowledge. I was in a state of fear that I could not describe. I had to learn how to give medications, home treatment, and the skills needed to use assistive equipment.

The triple repetition of “knowledge” conveys the intensity of her conviction that information was the most essential resource for empowerment. Her description of fear that she “could not describe” suggests an initial state of helplessness that was only resolved through acquiring practical medical knowledge. For this mother, learning to administer medications and operate assistive equipment transformed her from a frightened observer into an active agent in her child’s care. Another mother agreed:

Certainly, knowledge plays a role in achieving empowerment, which is an important demand…and this distinguishes some mothers from others in terms of talking and discussion. I want to be confident when searching for everything related to my daughter’s disability.

This mother’s observation that knowledge “distinguishes some mothers from others” introduces a social dimension to the concept of knowledge-based empowerment. Being well informed enables mothers to communicate confidently with professionals, challenge clinical decisions when necessary, and navigate service systems with greater efficiency. Knowledge, in this sense, functions as a form of social capital that determines the quality of a mother’s interactions with the professional world. One mother further described the transformative impact of acquiring practical clinical skills:

I need to have some skills to support my son, as waiting to reach the hospital could affect him… It is essential for us as mothers of children with severe disabilities… The day I changed his feeding tube by myself was a day I will never forget.

The feeding tube incident represented a critical turning point. Her ability to perform a clinical procedure independently eliminated her dependence on hospital-based care for routine but potentially life-threatening tasks. The emotional weight of this moment, described as “a day I will never forget,” reveals that empowerment through practical skills is not only about technical competence but also about the profound psychological shift from dependency to self-reliance in managing her child’s medical needs.

#### 3.1.3 Dimension three: resilience and well-being empowerment

This dimension addresses the psychological and emotional aspects of empowerment as experienced in a mother’s daily life. Mothers described empowerment as their ability to cope with the pressures of daily life, maintain psychological balance, rely on family support as an emotional anchor that strengthens their resilience, and achieve a state of inner contentment rooted in faith. Three sub-themes were identified.

##### 3.1.3.1 Empowerment as the ability to manage daily stress

In the context of SA, where mothers often bear the primary responsibility for the daily care of severe disabled children, coping with life’s pressures become a central part of their empowerment experience. In some cases, with limited institutional alternatives, empowerment is shaped by the ability to manage their daily lives and persevere despite increasing burdens. Within this framework, daily stress emerged as a constant and heavy burden, leading some mothers to define empowerment as their ability to alleviate or effectively manage stress. One stated:

Having a child with multiple severe disabilities is hard and constant work, and therefore it often limits the family’s day and activities; having a calm day makes me feel empowered, which is important.

Another mother added:

Empowerment is less daily stress. Before going to sleep, I feel anxious and afraid about managing the following day without stress.

These accounts are analytically significant because they define empowerment not through the presence of something positive, but through the absence of something negative. For these mothers, a calm and predictable day was not a baseline expectation but an aspiration. This reframing of empowerment as the absence of daily stress, rather than mere reduction, reflects the extreme and sustained nature of caregiving demands in the context of PIMDs and indicates that the current service provision fails to provide adequate relief from this burden.

##### 3.1.3.2 Empowerment as family cohesion and support

The intensive care requirements for PIMDs children often lead mothers to dedicate most of their time and effort to that child, potentially generating feelings of guilt toward their other children. In the context of SA, where mothers often bear the primary responsibility for daily care, this feeling is exacerbated by the weight of individual responsibility. In this regard, the involvement of siblings and family cohesion has emerged as supportive resources that alleviate psychological burden and contribute to empowering mothers by sharing responsibilities and restoring a degree of balance within family life. One mother said:

Sibling support plays a major role in my feeling of empowerment. It is an important aspect. This way, I can take care of both… We play and talk and help each other.

Her account constructs empowerment as a relational and distributed process within the family rather than as an individual maternal attribute. The sibling’s involvement enables her to fulfill her caregiving obligations to multiple children simultaneously, reducing the guilt associated with divided attention. Another mother described a family holiday as a turning point in her experience:

I did not imagine leaving the city of Riyadh or my neighborhood; the only outlet for me was the house garden. I will never forget that beautiful trip. My eldest and youngest sons were happy to carry her, choose the appropriate place, and play with her. It is essential to make all family members responsible for supporting [their children]. They all need to know how to take care of her if I am away.

This account has particular analytical weight. The mother’s initial confinement in her neighborhood and house garden reveals the degree of social isolation imposed by her caregiving responsibilities. The family trip represented not only a recreational activity but also a fundamental expansion of her physical and social worlds. Her insistence that all family members must learn to care for the child reflects a practical concern shared by most participants in this study: the fear of what would happen to their child in the event of the mother’s death or incapacity. For these mothers, distributing caregiving knowledge across the family is a form of futureproofing that constitutes a vital component of empowerment.

##### 3.1.3.3 Empowerment as inner satisfaction

This subtheme emerged as the most deeply held dimension of empowerment, linking it directly to the faith-based perspective that was distinctive of this sample of SA mothers. Although reaching this state often required time and psychological adjustment, it became a transformative force that reshaped how mothers viewed their child’s condition and their own role. One mother expressed:

It is a choice, not a punishment. The first stage of treatment is satisfaction with God’s gifts. So, choose satisfaction so that God will be pleased with you. God provides us with the best arrangements.

This mother’s framing of acceptance as a deliberate “choice” rather than passive resignation is analytically important. She positioned satisfaction with God’s will as the first step in the healing process, preceding any practical intervention. Her statement that God provides “the best arrangements” reflects a theological conviction that reinterprets the child’s disability not as a misfortune but as part of a divinely ordered plan. Another mother extended this spiritual reframing:

Some mothers are still living in this shell. What is in my daughter is not my fault. It is God’s choice. He could have created her perfect, but he created a disabled [child] in His wisdom. Contentment with His wisdom is strength…God chose me for a differently abled child because I am a good and strong mother, which is what the Quran says.

Her reference to “living in this shell” functions as a critique of mothers who remain trapped in grief or self-blame. She reframes her daughter’s condition as a divine decision made with wisdom and purpose and derives her identity as a strong mother directly from the Quranic teaching. This theological reinterpretation serves a dual psychological function: it removes personal guilt by attributing the child’s condition to God’s will and elevates the mother’s status by framing her selection for this role as evidence of her strength and worthiness. Other mothers reinforced this connection between faith and empowerment through the concept of divine reward:

With God, no effort or pain is wasted; it is a test, not an affliction. So, I will be rewarded with contentment and patience—if I am up to it. Oh God, make us able to withstand the test.

God wants me to be rewarded for every moment and effort. My path is laid with blessings all around me because I have a child from Heaven.

Both mothers reinterpreted their daily hardships within the framework of divine testing and rewards. The first mother’s distinction between a “test” and an “affliction” is theologically precise and psychologically consequential. Test implies purpose, growth, and eventual reward, whereas affliction implies meaningless suffering. The second mother’s description of her child as “from Heaven” and her caregiving path as “laid with blessings” represents a complete inversion of the deficit narrative commonly associated with severe disability. These faith-based accounts demonstrate that for SA mothers of PIMDs children, spiritual agency constitutes a culturally specific form of empowerment that may not be fully accommodated within some common conceptual frameworks of psychological empowerment.

#### 3.1.4 Dimension four: social-community empowerment

This dimension reflects the shift in mothers from individual experiences to a broader social sphere, where empowerment is viewed as social presence and voice. This is manifested through community recognition, participation in support networks, engagement in volunteer initiatives, and charitable work. In the SA context, which is witnessing an expansion of civil society initiatives and volunteer work alongside growing spaces for expression on social media platforms, this participation takes on a special dimension, representing a means for the mother to redefine their social roles and enhancing their presence beyond the confines of domestic care. Three sub-themes were identified.

##### 3.1.4.1 Empowerment through social media influencer and social networks

Several mothers described social media as their primary vehicle for empowerment, simultaneously functioning as a platform for advocacy, peer support, and personal transformation. One participant explained the following:

I communicated with the community and introduced them to my daughter. I showed them how to communicate with her despite her multiple disabilities… I went out to the community with confidence… This is the contentment that makes me feel empowered.

Her account revealed a multilayered empowerment process. Introducing her daughter to the community through social media served as a form of public visibility that challenged the social invisibility typically imposed on PIMDs children in SA society. Her use of the phrase “went out to the community with confidence” indicates that digital engagement preceded and enabled physical social participation, reversing the isolation that characterized her earlier experience. Another mother described a more dramatic personal transformation:

I was afraid of people’s view… But after I created my account. I became another person. My followers changed me, and I changed them. I share my travel journey with them and love reading their comments about how I have changed their lives with their children.

The shift from fear-induced social withdrawal to active community engagement represents a fundamental transformation of identity. Her statement that “my followers changed me, and I changed them” is analytically significant because it constructs empowerment as reciprocal and co-produced rather than individually achieved. The social media platform created a feedback loop in which sharing her experiences validated her journey while simultaneously supporting other parents. Her shift from fear to empowerment embodies the transformative potential of social media for parents of PIMDs children. Another mother identified the emotional support dimension of peer networks as follows:

Mothers who have children with severe disabilities are the most in need of additional emotional support… Professionals do not have time to discuss everything, but contact with other mothers helps a lot and empowers me. I have found friends—even sisters. We share experiences and motivate each other. It is support from a person with the same struggles.

Her observation that professionals lack the time for comprehensive emotional support reveals a gap in the formal service system filled with peer networks. Her description of fellow mothers as “friends—even sisters” indicates the depth of solidarity formed through shared experience of medical emergencies, hospital visits, and navigating unfamiliar resource systems. These relationships provide a form of experiential knowledge and mutual validation that professional support cannot fully replicate.

##### 3.1.4.2 Empowerment as community recognition and appreciation

Several mothers linked empowerment directly to receiving recognition and appreciation from their communities and professionals with whom they interacted. This recognition was not sought as praise but valued as an affirmation of their labor and competence in a role that is often socially invisible. One mother explained:

I got so many local awards as a mother who makes changes for children with disabilities and their parents… Every day I see them on my living room wall, and that is enough for me to feel successful and empowered.

The physical display of the awards on her living-room wall was not merely decorative. It functions as a daily visual affirmation of her identity as an effective advocate and caregiver, communicating this identity to family members and visitors alike. This public acknowledgement transformed her efforts from a private sacrifice into a publicly recognized contribution. Another mother described empowerment as follows:

I feel happy when the doctor praises me and says, ‘You are better than the nurses. You did a wonderful job.’ This is so empowering and satisfying for me.

Recognition by a medical authority is particularly significant, because it validates the clinical expertise that mothers develop through daily hands-on care. The doctor’s comparison with professional nurses acknowledges that these mothers have acquired practical competencies that rival those of trained healthcare workers. For mothers whose caregiving roles are often taken for granted or rendered invisible by the service system, such recognition constitutes a powerful form of professional affirmation that reinforces their sense of agency and worth.

##### 3.1.4.3 Empowerment as a shift toward voluntary charitable organizations

Mothers also conceptualized empowerment in relation to the role and orientation of voluntary and charitable organizations. Their accounts were largely critical, identifying a gap between the potential of these organizations and their current practices. One mother stated:

Charitable organizations have a different nature than what we aspire to… They are a source of strength, but unfortunately, we do not find the required support from them.

Her recognition of charitable organizations as a potential “source of strength” combined with her frustration at their failure to deliver meaningful support exposes a critical disconnect between organizational intent and family needs. Another mother elaborated on the nature of this shortcoming:

They request financial support from members more than having a clear plan or projects… Society is not accustomed to them due to their permanent isolation in shelters or at home, often bedridden, and because no project is available to introduce them to society positively.

This account is analytically important because it identifies two interconnected failures. The first is a strategic failure: the dominance of fundraising over program development means that these organizations lack concrete interventions that could improve families’ lives. The second is representational failure; the absence of projects that introduce PIMDs children to society perpetuates their invisibility and social isolation. Together, these mothers articulated a vision of empowerment in which charitable organizations would move from a traditional assistance model to a collaborative partnership that prioritizes family participation in decision-making and delivers sustainable needs-based interventions.

#### 3.1.5 Dimension five: civic empowerment and quality of life

This dimension explored the civic dimensions of empowerment as envisioned by mothers. Empowerment is not limited to daily caregiving but extends to controlling quality of life, participating in local decision-making, and striving to enrich their children’s lives by integrating them into social and recreational activities. In the SA context, the transformations associated with Vision 2030, with its focus on quality of life and enhanced community participation, have contributed to creating an environment that encourages this civic understanding of empowerment and facilitates its shift from a private family experience to a broader social presence. Three sub-themes were identified.

##### 3.1.5.1 Empowerment as control over quality of life

Some mothers conceptualized empowerment as the ability to exercise meaningful control over the direction and quality of their and their children’s lives. This control has often been described in contrast to the constraints imposed by professionals and bureaucratic systems. One mother framed her frustration as a question:

The power to become someone who gains benefits, achieves what I desire… Why are they controlling my dream?

Her use of the word “dream” is significant because it locates empowerment not only in the practical domain of service access but also in the aspirational domain of personal fulfillment. The question, “Why are they controlling my dream?” is directed at a system that she perceives as restricting her agency rather than supporting it. Another mother expressed a similar aspiration:

Empowerment is the main requirement; it is taking initiative without obstacles that waste time and effort. Let my strength and power help me get what I need.

Her framing of empowerment as “taking initiative without obstacles” positions the problem not in the mother’s capacity but in the systemic barriers that prevent her existing strength from being effective. A third mother offered a concrete example of meaningful choices in practice.

Empowerment provides various options, and I am not forced to just do what I am told or take what is available… It is having a life… I was given three hospitals as an option and made my own decision about where my son would have a follow-up.

Her definition of empowerment as “having a life” and “not being forced” captures a core aspiration shared across many participants. The example of choosing among three hospitals demonstrates that empowerment is produced not merely through access to services but also through the availability of alternatives and the freedom to exercise judgment. For mothers accustomed to receiving directives rather than options, this experience of autonomous decision-making represented a qualitative shift in their relationship with the service system.

##### 3.1.5.2 Empowerment as involvement in local policymaking

Mothers also connected empowerment to their engagement with the policy and regulatory frameworks that governed disability services. Their accounts revealed their frustration with the inadequacy of existing policies and their desire to participate directly in shaping them. One mother explained:

I read some regulations, and they seemed so general, unclear, and unrelated to my situation. It did not make me feel empowered at all.

Another offered a comparative perspective:

I learned about an Australian system through a scholarship, and I was impressed by the details mentioned in it and how it is so linked to our life.

These two accounts, when placed together, expose a gap between the specificity required by families with PIMDs children and the generality of the current SA regulations. The first mother’s experience of policies that were “unrelated to my situation” indicates that the existing legislation does not account for the particular complexities of PIMDs. The second mother’s admiration for the Australian system was grounded in its detailed connection to daily lived experiences, providing a model of what policy responsiveness could look like. This awareness fueled the desire for direct participation:

Just thinking about being a policymaker empowers me. I would be able to communicate through it.

Her statement that she would “communicate through” policymaking is analytically significant. She does not view policy participation as traditional advocacy or protest but as a communication channel through which her daily experiences and needs could be translated into an institutional language. She positions direct involvement in decision-making as a mechanism for reshaping the discourse on disability policy from within, ensuring that the perspectives of those closest to the issue inform the formulation of solutions.

##### 3.1.5.3 Empowerment as entertainment and changing the child’s lifestyle

Some mothers associated empowerment with enabling their children to access stimulation, enjoyment, and active participation in family life. Their accounts revealed that the daily routines of PIMDs children were often reduced to basic care functions, with limited opportunities for play, recreation, or social engagement. One mother described:

There is nothing suitable outside. I often rely on myself to provide her with entertainment at home, such as a small swimming pool or toy trolley… Her siblings may share with her, but the place is tiny. Her time is spent sleeping, eating, and cleaning.

Another mother expressed a similar frustration:

Outside activities are not suitable for her at all. She cannot share anything with her siblings, which is why she is usually left at home. Her day is a boring routine.

Both accounts reveal that the absence of accessible entertainment and recreation is not a minor inconvenience but a structural barrier to the child’s quality of life and social inclusion. The first mother’s description of her daughter’s day as limited to “sleeping, eating, and cleaning” exposes the reduction of a child’s life to basic physiological maintenance, with no space for stimulation, play, or developmental enrichment. The second mother’s observation that her daughter “cannot share anything with her siblings” reveals how inaccessible environments produce social exclusion within the family unit itself. For both mothers, empowerment referred to moving beyond survival-oriented care to a model that included recreation and shared family experiences as essential components of their child’s life.

### 3.2 Constructing the dimensions of lived-experience-based empowerment

The identification of the dimensions of what this study terms “lived-experience-based empowerment” (LEBE) was based on an inductive analysis of data. The five dimensions emerged from recurring patterns in mothers’ experiences and distinct areas where empowerment manifests in their daily lives. These dimensions were not presupposed but rather formed by synthesizing detailed concepts extracted from interviews and organizing them into higher levels of abstraction. The results reveal that empowerment, as perceived by mothers, is not understood simply as access to resources or services but as an ongoing process that reshapes how they approach their daily roles and responsibilities. It is not a static state but a dynamic experience shaped by the continuous interaction between mother and child, surrounding services, and a broader social context.

The analysis reveals that this empowerment does not arise solely from the mothers’ individual characteristics in isolation from the context but is rather shaped through interconnected relationships encompassing the mother’s characteristics, child’s needs, responses of the support system, and broader societal factors. A sense of empowerment is linked to a mother’s ability to interact with and influence these different levels, thereby enhancing her child’s quality of life and her role as a primary caregiver. Thus, it becomes clear that lived-experience empowerment is a multidimensional process that operates simultaneously at both the personal and systemic levels. It transforms from an abstract concept into a daily practice shaped by the interaction between subjective experience and surrounding institutional and social responses.

## 4 Discussion

The findings reveal that the concept of LEBE, as perceived by mothers of PIMDs children, is not formed as a static state or a direct result of institutional intervention but rather as a dynamic process that gradually crystallizes through daily situations and accumulated experiences. The sense of empowerment does not stem solely from formally granting authority or providing services but from the accumulation of practical experience embodied in daily care practices and the resulting knowledge and ability to influence. From this perspective, this study discusses the place of this understanding within the broader theoretical framework of disability studies, exploring its intersections with the assumptions of prevailing disability models and the points of agreement and disagreement between them.

### 4.1 Mothers’ perceptions of empowerment intersect with models of disability

#### 4.1.1 Divergent conceptions of empowerment from traditional disability models

First, the charity model of disability portrays disabled people as passive victims who deserve regret and philanthropic aid and is reinforced by narratives of tragedy, sympathy, and despair (Sofokleous and Stylianou, 2023; Shakespeare, 1994), sustaining stereotypes of helplessness and dependency (Mugabi, 2017; Retief and Letšosa, 2018). LEBE departs from this view by presenting empowerment as stable right support rather than occasional charitable help. The results reveal how mothers called for voluntary associations to move away from emergency grants and seasonal aid toward sustainable and professional services that engaged them as active partners rather than powerless recipients. These findings are consistent with previous work that highlights that pity weakens positive identity and generates feelings of inferiority, while support based on appreciation, empowerment, and respect is preferred (Dunn and Burcaw, 2013); that disabled people experience pity as a form of “social superiority” that keeps them in an inferior position and maintains barriers (Deal, 2003); and that pity is a psychologically damaging attitude that casts people as “helpless and needy” instead of recognizing their abilities and rights (Olkin, 2012).

Second, medical-psychological models that locate the problem within the child blame parents or treat them as secondary to specialist authority (Haegele and Hodge, 2016; Laiyan, 2024; Lawson and Beckett, 2021). By contrast, LEBE adopted by mothers of PIMDs children is based on acquiring experience and developing specialized knowledge about their children’s condition. This represents a clear shift from the negative role imposed by the medical-psychological models, which confines knowledge and experience to specialists and marginalizes the role of parents as active partners in understanding their children’s condition and making decisions related to their care. As explained by one of the participants, “Knowledge…is…foundation…[that] distinguishes some mothers from others in terms of talking and discussion.” Thus, mothers seek to acquire practical knowledge that restores their ability to understand, make decisions, and interact more effectively with service providers. The mothers also linked empowerment to controlling their children’s quality of life and their own lives. One mother said, “The power to become someone who gains benefits achieves what I desire…Why are they controlling my dream?*”* She refused a role that confined the agency to specialists. LEBE places mothers at the center of organizing care and daily decisions for their children and does not confine expertise to professionals. Previous studies support this view, as parents of PIMDs children often hold unique and continuous knowledge of their child and interpret communication cues and pain more accurately than do professionals (Aim et al., 2023; Kruithof et al., 2020). De Geeter et al. (2002) argued that professionals should treat “parents as experts” by valuing their experience, using their input in care planning, and keeping them informed about the child’s progress.

#### 4.1.2 Rejection of empowerment conceptions in light of the normalization model

The normalization model is a transitional, intermediate model between traditional and modern positive models. Thus, the LEBE revealed in this study gives value to human diversity, unlike the normalization model, which assumes that the goal is to move the child closer to prevailing norms. The mothers described their children’s differences as a part of human nature and linked empowerment to the active inclusion of their children in family and community life through suitable and safe recreational activities. One mother stated, “She cannot share anything with her siblings, which is why she is usually left at home. Her day is a boring routine.*”* These findings highlight the need for more accessible leisure opportunities. Mothers also reported empowerment through confident engagement on social media, positive influence on society, and challenging disability stereotypes by public presence and sharing of experiences, as in the comment “I went out to the community with confidence.*”* They described a humanistic view of their children’s differences by rejecting fatalistic beliefs or those that assign blame. For example, one mother said, “God chose me for a differently abled child because I am a good and strong mother, which is what the Quran says.*” She* linked empowerment to emotional and spiritual strength based on acceptance of herself and her child and not on conformity with external standards of normality. Another mother explained that home-based supports and equipment helped her child “maintain normal living.” “*Normal”* here differs from the normalization model, which presents empowerment as actions aimed at helping a person conform to socially accepted standards (Kumar et al., 2015; Race, 2002; Aljaser, 2020; Winance, 2007; Chappell, 1992; Zaks, 2023), whereas the LEBE refers to adapting to the environment and providing tools that allow children to live and participate in their own way.

#### 4.1.3 Conceptions of empowerment considering contemporary disability models: intersection and transcendence

First, the social model of disability posits that disability arises from environmental barriers rather than from an individual’s physical or mental limitations and seeks to reform society rather than the individual (Oliver, 1996; Shakespeare, 2006). In other words, people are not “disabled” because of their physical or mental differences, but because of a society that fails to remove the barriers that limit their opportunities for equal and effective participation in various aspects of life. This is exemplified elegantly with the LEBE in several examples, such as “Empowerment is streamlined; no need to work hard to access service*”* and “Cooperation is empowering.*”* “The delay in providing comprehensive support will affect them more,” where empowerment defines an integrated coordination of services and support that enables mothers to access various resources and systems easily, reduces bureaucratic complexities, and enhances their ability to meet their children’s needs comprehensively, sustainably, and promptly. This aligns with findings of previous studies indicating that PIMDs families face a compound burden that extends beyond the disability itself, including unsupported responsibilities in managing complex clinical tasks (Smith et al., 2015), transportation difficulties that hinder family integration (McKenzie et al., 2021), and poor coordination as additional structural obstacles (Lahaije et al., 2023), necessitating the removal of obstacles by creating suitable environments and facilitating services. However, even though the LEBE intersects with the social model in its focus on removing barriers—which the social model assumes results from these obstacles rather than placing responsibility on the individual—it goes further, recognizing that removing barriers is insufficient. Instead, it focuses on the dynamic relationship between individuals and their environment and how this environment empowers them to make decisions, act, and thereby enhance their capacity for action and influence.

Second, the rights-based disability model endorses advocacy and accountability, an empowerment approach that seeks to remove barriers, ensure accessibility, and promote participation (Bickenbach, 2001; Degener, 2017; Eaton et al., 2021; Lawson and Beckett, 2021; Szlamka et al., 2022). Scholars such as Banach et al. (2010), Itzhaky and Schwartz (2001), Koren et al. (1992), and Wakimizu et al. (2011) have linked empowerment to parents’ advocacy efforts and formal mechanisms that hold systems accountable. The LEBE in this study presents a different picture. The mothers rarely framed empowerment as fighting for rights; they defined it as the ability to live and make decisions without constant obstacles. One mother described empowerment as “it is taking initiative without obstacles that waste time and effort.*”* This definition centers on control over one’s private life and the freedom to choose what is best within the available systems. Another mother stated, “Empowerment is crucial, providing what it should provide.*”* She linked empowerment to a welcoming and respectful environment in which services functioned smoothly, communication with providers was open, and mothers’ experiences were valued. In this sense, empowerment depends on systems that anticipate needs and solve problems proactively, rather than on parents’ continual advocacy. This interpretation corresponds with studies indicating that repeated advocacy can be exhausting, frustrating, and sometimes damaging to parent-professional relationships (Trainor, 2010; Rehm et al., 2013; Burke et al., 2019; Burke et al., 2021; Burke and Hodapp, 2016; Shapiro et al., 2004; Boxall et al., 2002). Therefore, LEBE shifts its focus from empowerment through struggle to empowerment through responsive, cooperative services that reduce the need for adversarial action.

Third, the LEBE identified in this study shares core ideas with the affirmation model of disability, which rejects the assumption that disability is inherently negative or fixed and instead treats it as a basis for positive identity grounded in lived experience (Cameron, 2015; Swain and French, 2000). The affirmation model presents impairment as an integral part of a person’s identity and calls for ordinary and respectful treatment of differences in language and practice (Cameron, 2008; Cameron, 2023; Aljaser, 2020). In this study, the mothers of PIMDs children supported this orientation by describing empowerment through visibility, solidarity, and public presence. One mother stated, “I was encouraged to present my experiences. I went out to the community with confidence,*”* linking empowerment to confidence in appearing as the mother of a severely disabled child in community spaces and social media. Mothers used these platforms to share their experiences, challenge stigmas, and exchange practical strategies with others in similar situations. However, the LEBE moved beyond the affirmation model’s focus on identity and pride. It adds a practical strand, in which empowerment also depends on access to specialized knowledge, the ability to adjust to home and service environments, and the capacity to make effective decisions in daily care. Thus, the LEBE connects positive identity and acceptance with everyday competence in supporting PIMDs children, demonstrating that affirmation and empowerment can operate together in mothers’ lives.

#### 4.1.4 Reframing empowerment by considering the religious model of disability

The LEBE in this study also engages in a religious and faith-based understanding of disabilities. Religious and faith perspectives describe disability as being linked to divine wisdom, mercy, and spiritual testing, rather than medical or social causes (Aljaser, 2020; Bazna and Hatab, 2005; Bennett and Volpe, 2018; Ghaly, 2016; Retief and Letšosa, 2018; Schuelka, 2013). These models suggest that impairment can function as a divine test offering opportunities for spiritual growth (Black, 1996). The mothers of PIMDs children in this study echoed this orientation, albeit in a reflective and empowering manner. One mother said, “With God, no effort or pain is wasted; it is a test, not an affliction.*”* Another described her experience by stating, “My path is laid with blessings all around me because I have a child from Heaven.” These accounts rejected guilt-and-punishment narratives and presented faith as a source of acceptance and dignity for both mothers and their children. Mothers framed their children as trusted, but only as a sign of moral failure. Simultaneously, the LEBE indicated that this inner content did not lead to passive acceptance. Spiritual peace has become a motivation for action and responsibility, strengthening mothers’ capacity to make decisions, organize care, and construct positive meanings in daily life. Trust in God’s plans served as a foundation for empowerment, supporting resilience and purposeful caregiving within the family environment.

### 4.2 Re-examining the concept of empowerment in light of mothers’ lived experience

A critical comparison of mothers’ perceptions of empowerment and different models of disability reveals that these perceptions do not fully conform to any existing model but rather interact with them selectively and critically. The results reveal a clear divergence from traditional models that tend to reduce disability to impairment or the need for care and a rejection of the normalization approach that focuses on aligning the child with prevailing norms. Conversely, mothers’ perceptions revealed important intersections with contemporary models of disability, particularly in recognizing environmental barriers, the importance of rights, building a positive identity, and the role of meaning and faith in dealing with disability. However, the findings of this study demonstrate that mothers’ experience of empowerment transcends the boundaries of these models by emphasizing the lived, everyday nature of empowerment and its interconnectedness across multiple levels, including the individual, the family, services, and the community. This interaction highlights that empowerment is not an abstract theoretical concept but a complex and evolving process shaped by context and experience, which necessitates a deeper interpretive understanding rooted in the mothers’ own experiences.

Based on the findings of this study, empowerment can be understood as a multidimensional, contextual process arising from the ongoing interaction among a mother’s characteristics, her child’s needs, the nature of her family relationships, the support services she accesses, and the broader social context in which she lives. Empowerment is not viewed here as a static state or an isolated, individual achievement but rather as a daily practice rooted in lived experiences, shaped through interaction with challenges, the construction of meaning, and the development of coping and support strategies. This understanding reflects that empowerment is realized when more responsive and inclusive environments are available, enabling mothers to fulfill their roles effectively and foster a sense of competence, meaning, and ability to influence their own lives and those of their children, moving beyond the reduction of empowerment to simply providing resources or granting authority.

Thus, maternal empowerment can be understood as “a multifaceted process arising from the ongoing interaction between the mother’s characteristics, her child’s needs, the surrounding social environment, and broader systemic factors. Empowerment is not viewed as a one-dimensional experience, but rather as an interactive practice shaped by daily relationships and experiences, contributing to a responsive and supportive environment that enhances mothers’ ability to live meaningful lives. This understanding reflects that empowerment is not simply about providing opportunities or resources but is embodied in spontaneous and automatic daily practices stemming from a mother’s sense of responsibility and competence, driven by a desire to live meaningfully without waiting for authorization from any authority.” This interpretive understanding integrates the five dimensions revealed by the findings, illustrating how mothers’ individual experiences are woven into family, service, and community contexts to shape their empowerment experiences. Thus, this definition serves as an interpretive summary of the discussion, reflecting how empowerment is reinterpreted from mothers lived experiences.

## 5 Limitations and future research perspectives

This study did not focus on a specific age group of children or the specific demographic characteristics of mothers, such as employment or economic status, which contributed to the diversity of experiences and a richer dataset. However, this diversity may limit the exploration of subtle differences between various groups and reduce the translatability of the findings to more homogeneous populations, as empowerment needs may vary depending on factors such as the child’s developmental stage, mother’s employment status, and family’s economic resources. Furthermore, the study relied on individual interviews with a limited number of participants in specific sociocultural contexts. This reflects the nature of qualitative research, which aims for an in-depth understanding rather than statistical generalization. Therefore, the results should be interpreted as contextual representations of the participants’ experiences and not as sweeping generalizations for all mothers of PIMDs children. Therefore, the study recommends that future research should be conducted with more homogeneous samples (such as only working mothers, only non-working mothers, or within specific age groups of children), along with the adoption of longitudinal qualitative designs that explore the evolution of the concept of empowerment across different stages of a child’s and family’s lives.

## 6 Conclusion and practical implications

This study makes three major contributions to the literature. First, it helps fill the knowledge gap by focusing on mothers of PIMDs children, a group that remains underrepresented in the empowerment literature compared to those with less severe disabilities. Second, it makes a conceptual contribution by highlighting empowerment as a dynamic and multidimensional lived experience shaped by the ongoing interaction between the mother’s characteristics, the child’s needs, family relationships, the service system, and the broader social context. Thus, this study deepens the existing understanding of empowerment by reinterpreting it from the perspective of mothers’ daily experiences without seeking to offer an alternative model. Third, the study adds a contextual and applied dimension by analyzing the experience of empowerment in SA society, highlighting the impact of service infrastructure and social factors in shaping its meaning and providing insights that can benefit policymakers and service providers in developing practices that are more responsive to the realities of this group.

## Data Availability

The original contributions presented in the study are included in the article/supplementary material, further inquiries can be directed to the corresponding author.

## References

[ref1] Aim M. Rousseau M. Hamouda I. Anzola A. B. de Villemeur T. B. Milh M. . (2023). Parents’ experiences of parenting a child with profound intellectual and multiple disabilities in France: a qualitative study. Health Expect. 27, 26–52. doi: 10.1111/hex.13910 PMC10757136 37932892

[ref2] Alahmad M. R. M. (2017) The effectiveness of two counseling programs based on reality therapy and logotherapy in developing planning and psychological empowerment among mothers of children with intellectual disabilities [Doctoral dissertation]. Jordan: Mutah University. Available online at: http://search.mandumah.com/Record/957151

[ref3] Aldalbahi K. (2018). Social support in its relationship with psychological empowerment in mothers of children with intellectual disabilities in special education programs in Dawadmi. J. Humanit. Soc. Sci. 29, 161–199.

[ref4] Aljaser K. (2017). Identifying the interactive communication ability of children with profound and multiple learning disabilities: the intensive interactive approach. Glob. J. Spec. Educ. Serv. 5, 106–125.

[ref5] Aljaser K. (2020). School Leaders’ Perceptions of Differing Intellectual Abilities in the Context of Inclusive School Culture in the Kingdom of Saudi Arabia: Transformational Leadership Theory in Disability Studies. Sheffield: University of Sheffield.

[ref6] Aljaser K. (2025). Contemporary special education in Kingdom of Saudi Arabia: conceptualization and service provision for profound and multiple intellectual disabilities. Egypt. J. Educ. Sci. 5:759. doi: 10.21608/ejes.2025.479759

[ref7] Almosaadah G. (2010). The effect of a Training Program Based on Psychological Empowerment on Improving Self-Compassion and marital Satisfaction Among Mothers of Handicapped Children. Jordan: Hashemite University.

[ref8] Alnemr A. (2020). The relative contribution of mothers’ psychological empowerment in predicting adjustment behaviour of their children with mild mental disabilities. J. Spec. Needs Sci 3, 1357–1408. doi: 10.21608/jshm.2020.47795.1072

[ref9] Alsem M. W. van Meeteren K. M. Verhoef M. Schmitz M. J. W. M. Jongmans M. J. Meily-Visser J. M. A. . (2017). Co-creation of a digital tool for the empowerment of parents of children with physical disabilities. Res. Involv. Engagem. 3:26. doi: 10.1186/s40900-017-0079-6, 29238612 PMC5724239

[ref10] Anderson G. J. Arsenault N. (2002). Fundamentals of Educational Research (A Pragmatic Approach). 2nd Edn London: Routledge Falmer.

[ref11] Asran M. (2018). The effectiveness of a counseling program based on positive psychological support in improving the psychological empowerment of mothers and its impact on the adaptive behavior of their mentally retarded children. J. Spec. Educ. 25, 137–239.

[ref12] Banach M. Iudice J. Conway L. Couse L. J. (2010). Family support and empowerment: a post autism diagnosis support group for parents. Soc. Work Groups 33, 69–83. doi: 10.1080/01609510903437383

[ref13] Baumstarck K. Hamouda I. Iline N. El Ouazzani H. Fouladvand M. Loukkal S. . (2025). Quality of life among individuals with profound intellectual and multiple disabilities: crossed perspectives of institutional caregivers and parents. Health Qual. Life Outcomes 23:34. doi: 10.1186/s12955-025-02434-3, 41053826 PMC12502390

[ref14] Bazna M. S. Hatab T. A. (2005). Disability in the Qur’an: the Islamic alternative to defining, viewing, and relating to disability. J. Relig. Disabil. Health 9, 5–27. doi: 10.1300/J095v09n01_02

[ref15] Bennett J. M. Volpe M. A. (2018). Models of disability from religious tradition: introductory editorial. J. Disabil. Relig. 22, 121–129. doi: 10.1080/23312521.2018.1482134

[ref16] Bickenbach J. E. (2001). “Disability human rights, law and policy,” in Handbook of Disability Studies, eds. Albrecht M. Seelman K. Bury M. (Thousand Oaks: Sage Publications), 565–584.

[ref17] Black K. (1996). A Healing Homiletic: Preaching and Disability. Nashville: Abingdon Press.

[ref18] Boxall K. Jones M. Smith S. (2002). “Advocacy and parents with learning difficulties: “even when you have got an advocate social services still always do what’s easiest for them,”,” in Learning Disability: A Social Approach, ed. Race D. G. (London: Routledge), 209–226.

[ref19] Braun V. Clarke V. (2006). Using thematic analysis in psychology. Qual. Res. Psychol. 3, 77–101. doi: 10.1191/1478088706qp063oa

[ref20] Burke M. M. Hodapp R. M. (2016). The nature, correlates, and conditions of parental advocacy in special education. Exceptionality 24, 137–150. doi: 10.1080/09362835.2015.1064412

[ref21] Burke M. M. Lee C. E. Rios K. (2019). A pilot evaluation of an advocacy programme on knowledge, empowerment, family–school partnership, and parent well-being. J. Intellect. Disabil. Res. 63, 969–980. doi: 10.1111/jir.12613 30815933

[ref22] Burke M. M. Rossetti Z. Aleman-Tovar J. Rios K. (2021). Examining the relation between empowerment and civic engagement among parents of individuals with intellectual and developmental disabilities. J. Appl. Res. Intellect. Disabil. 34, 1569–1581. doi: 10.1111/jar.12901, 33998114

[ref23] Cameron C. (2008). “Further towards an affirmation model,” in Disability Studies: Emerging Insights and Perspectives, eds. Campbell T. Fontes F. Hemingway L. Soorenian A. Till C. (Leeds: The Disability Press).

[ref24] Cameron C. (2015). Turning experience into theory: the affirmation model as a tool for critical praxis. Soc. Work. Soc. Sci. Rev. 17, 108–121. doi: 10.1921/swssr.v17i3.802

[ref25] Cameron C. (2023). Some things never seem to change: further towards an affirmation model. Disabil. Soc. 39, 1890–1895. doi: 10.1080/09687599.2023.2295799

[ref26] Chappell A. L. (1992). Towards a sociological critique of the normalisation principle. Disabil. Handicap Soc. 7, 35–51. doi: 10.1080/02674649266780041

[ref27] Clifton S. Cooper E. Bourke J. Connor S. Denton S. Dominish B. . (2025). A framework for disability lived expertise. Evid Policy 1–21. doi: 10.1332/17442648Y2025D000000069 41073073

[ref28] Cohen L. Manion L. Morrison K. (2010). Research Methods in Education. 6th Edn London: Routledge.

[ref29] Damra L. (2015). Level of empowerment among families of children with disabilities in Jordan. J. Spec. Educ. Rehabil. 7, 1–29.

[ref30] Dan B. (2020). Disability and empowerment. Dev. Med. Child Neurol. 62, 536–536. doi: 10.1111/dmcn.14511 32249940

[ref31] De Geeter K. I. Poppes P. Vlaskamp C. (2002). Parents as experts: the position of parents of children with profound multiple disabilities. Child Care Health Dev. 28, 443–453. doi: 10.1046/j.1365-2214.2002.00294.x, 12568473

[ref32] Deal M. (2003). Disabled people’s attitudes toward other people with impairments: a hierarchy of impairment. Disabil. Soc. 18, 897–910. doi: 10.1080/0968759032000127317

[ref33] Degener T. (2017). “A new human rights model of disability,” in The United Nations Convention on the Rights of Persons with Disabilities. Eds. V. Della Fina, R. Cera, G. Palmisano, (Cham: Springer International Publishing), 41–59.

[ref34] Dempsey I. Dunst C. J. (2004). Helpgiving styles and parent empowerment in families with a young child with a disability. J. Intellect. Develop. Disabil. 29, 40–51. doi: 10.1080/13668250410001662874

[ref35] Dunn D. S. Burcaw S. (2013). Disability identity: exploring narrative accounts of disability. Rehab. Psychol. 58, 148–157. doi: 10.1037/a0031691, 23437994

[ref36] Eaton J. Carroll A. Scherer N. Daniel L. Njenga M. Sunkel C. . (2021). Accountability for the rights of people with psychosocial disabilities: an assessment of country reports for the convention on the rights of persons with disabilities. Health Hum. Rights 23, 175–189. 34194211 PMC8233018

[ref37] Federici S. Meloni F. Brogioni A. Lo Presti A. (2008). The disability models in the perspective of parents, teachers, and special needs educators: a qualitative data analysis. Open Educ. J. 1, 37–48. doi: 10.2174/1874920800801010037

[ref38] Fischer P. Goodley D. (2007). The linear medical model of disability: mothers of disabled babies resist with counter-narratives. Sociol. Health Illn. 29, 66–81. doi: 10.1111/j.1467-9566.2007.00518.x, 17286706

[ref39] Fujioka H. Wakimizu R. Tanaka R. Ohto T. Ieshima A. Yoneyama A. . (2015). Empirical study on the empowerment of families raising children with severe motor and intellectual disabilities in Japan: the association with positive feelings towards child rearing. Health 7, 1725–1740. doi: 10.4236/health.2015.712188

[ref40] Geuze L. Goossensen A. (2021). Exploring the experiences of Dutch parents caring for children with profound intellectual and multiple disabilities: a thematic analysis of their blogs. Glob. Qual. Nurs. Res. 8:23333936211028170. doi: 10.1177/23333936211028170, 34263011 PMC8252404

[ref41] Ghaly M. (2016). Disability in the Islamic tradition. Relig. Compass 10, 149–162. doi: 10.1111/rec3.12202

[ref42] Gona J. K. Newton C. R. Hartley S. Bunning K. (2018). Persons with disabilities as experts-by-experience: using personal narratives to affect community attitudes in Kilifi, Kenya. MC Int. Health Hum. Rights 18, 1–12. doi: 10.1186/s12914-018-0158-2, 29739403 PMC5941597

[ref43] Gray D. E. (2014). Doing Research in the Real World. 3rd Edn London: Sage Publications.

[ref44] Haegele J. A. Hodge S. (2016). Disability discourse: overview and critiques of the medical and social models. Quest 68, 193–206. doi: 10.1080/00336297.2016.1143849

[ref45] Halat E. (2012). Family empowerment indicators as a comprehensive quality standard in civil-society institutions for the care of disabled children. Child. Educ. Mag 12, 17–76.

[ref46] Han K. S. Yang Y. Hong Y. S. (2018). A structural model of family empowerment for families of children with special needs. J. Clin. Nurs. 27, e833–e844. doi: 10.1111/jocn.14195, 29193505

[ref47] Huang Y. P. Kellett U. M. St John W. (2010). Cerebral palsy: experiences of mothers after learning their child’s diagnosis. J. Adv. Nurs. 66, 1213–1221. doi: 10.1111/j.1365-2648.2010.05270.x, 20546355

[ref48] Hui M. K. Au K. Fock H. (2004). Empowerment effects across cultures. J. Int. Bus. Stud. 35, 46–60. doi: 10.1057/palgrave.jibs.8400076

[ref49] Itzhaky H. Schwartz C. (2001). Empowerment of parents of children with disabilities: the effect of community and personal variables. J. Fam. Soc. Work. 5, 21–36. doi: 10.1300/J039v05n01_03

[ref51] Kalleson R. Jahnsen R. Østensjø S. (2019). Empowerment in families raising a child with cerebral palsy during early childhood: associations with child, family and service characteristics. Child Care Health Dev. 46, 19–27. doi: 10.1111/cch.12716, 31503355

[ref52] King Saud University Research Ethics Policy (2015). Ethical Guidelines for Conducting Research Involving Human Participants. Riyadh: King Saud University.

[ref53] Koren P. E. DeChillo N. Friesen B. J. (1992). Measuring empowerment in families whose children have emotional disabilities: a brief questionnaire. Rehabil. Psychol. 37, 305–321. doi: 10.1037/h0079106

[ref54] Kruithof K. Willems D. van Etten-Jamaludin F. Olsman E. (2020). Parents’ knowledge of their child with profound intellectual and multiple disabilities: an interpretative synthesis. J. Appl. Res. Intellect. Disabil. 33, 1141–1150. doi: 10.1111/jar.12740, 32367663 PMC7687241

[ref55] Kumar A. Singh R. R. Thressiakutty A. T. (2015). Normalization vs. social role valorization: similar or different? Int. J. Spec. Educ 30, 71–78. doi: 10.2139/ssrn.3565830

[ref56] Lahaije S. T. A. Luijkx J. Waninge A. van der Putten A. A. J. (2023). Well-being of families with a child with profound intellectual and multiple disabilities. Res. Pract. Persons Severe Disabil. 48, 63–78. doi: 10.1177/15407969231173916

[ref57] Laiyan M. T. (2024). Empowerment model for persons with disabilites: application village inclusion for incumbent disability in the village Mangliawan Pakis District, Malang regency. KnE Life Sci. 8, 432–440. doi: 10.18502/kls.v8i2.17354

[ref58] Lawson A. Beckett A. E. (2021). The social and human rights models of disability: towards a complementarity thesis. Int. J. Hum. Rights 25, 348–379. doi: 10.1080/13642987.2020.1783533

[ref59] Luijkx J. van der Putten A. A. J. Vlaskamp C. (2017). Time use of parents raising children with severe or profound intellectual and multiple disabilities. Child Care Health Dev. 43, 518–526. doi: 10.1111/cch.12446, 28156014

[ref61] Luitwieler N. Luijkx J. Salavati M. Van der Schans C. P. Van der Putten A. J. Waninge A. (2021). Variables related to the quality of life of families that have a child with severe to profound intellectual disabilities: a systematic review. Heliyon 7:e07372. doi: 10.1016/j.heliyon.2021.e07372, 34401546 PMC8353312

[ref62] Mahmic S. Kern M. L. Janson A. (2021). Identifying and shifting disempowering paradigms for families of children with disability through a systems-informed positive psychology approach. Front. Psychol. 12:663640. doi: 10.3389/fpsyg.2021.663640, 35002821 PMC8734639

[ref63] Matthews B. Ross L. (2010). Research Methods: A Practical Guide for the Social Sciences. Harlow: Pearson Education.

[ref64] McCann D. Bull R. Winzenberg T. (2012). The daily patterns of time use for parents of children with complex needs: a systematic review. J. Child Health Care 16, 26–52. doi: 10.1177/1367493511420186, 22308543

[ref66] McKenzie J. A. Kahonde C. Mostert K. Aldersey H. M. (2021). Community participation of families of children with profound intellectual and multiple disabilities in South Africa. J. Appl. Res. Intellect. Disabil. 34, 525–536. doi: 10.1111/jar.12818 33040428

[ref67] Monje-Amor A. Xanthopoulou D. Calvo T. Abeal Vázquez J. (2021). Structural empowerment, psychological empowerment and work engagement: a cross-country study. Hum. Resour. Manag. J. 31, 668–685. doi: 10.1111/1748-8583.12325

[ref68] Mugabi I. (2017). The legality of the charitable model of disability under corporate social responsibility (CSR). SSRN. [Preprint]. doi: 10.2139/ssrn.3051490

[ref69] Murray M. M. Handyside L. M. Straka L. A. Arton-Titus T. V. (2013). Parent empowerment: connecting with preservice special-education teachers. Sch. Community J. 23, 145–168.

[ref70] Nachshen J. S. Minnes P. (2005). Empowerment in parents of school-aged children with and without developmental disabilities. J. Intellect. Disabil. Res. 49, 889–904. doi: 10.1111/j.1365-2788.2005.00721.x, 16287478

[ref71] Nakken H. Vlaskamp C. (2007). A need for a taxonomy for profound intellectual and multiple disabilities. J. Policy Pract. Intellect. Disabil. 4, 83–87. doi: 10.1111/j.1741-1130.2007.00104.x PMC11582349 39582792

[ref72] Oliver M. (1996). Understanding Disability: From Theory to Practice. London: Macmillan Education.

[ref73] Olkin R. (2012). What Psychotherapists should know about Disability. New York, NY: The Guilford Press.

[ref74] Race D. (2002). “The normalisation debate—time to move on,” in Learning Disability: A Social Approach, ed. Race D. G. (Abingdon: Routledge), 209–226.

[ref75] Rankin J. Regan S. (2004). Meeting complex needs in social care. Housing Care Support 7, 4–8. doi: 10.1108/14608790200400016

[ref76] Reeder J. Morris J. (2021). Becoming an empowered parent: how do parents successfully take up their role as a collaborative partner in their child’s specialist care? J. Child Health Care 25, 110–125. doi: 10.1177/1367493520910832, 32141316

[ref77] Rehm R. S. Fisher L. T. Fuentes-Afflick E. Chesla C. A. (2013). Parental advocacy styles for special education students during the transition to adulthood. Qual. Health Res. 23, 1377–1387. doi: 10.1177/1049732313505915, 24062419 PMC4028223

[ref78] Retief M. Letšosa R. (2018). Models of disability: a brief overview. HTS Teol. Stud. 74, 1–9. doi: 10.4102/hts.v74i1.4738

[ref79] Rousseau M.-C. Baumstarck K. Khaldi-Cherif S. Brisse C. Felce A. Moheng B. . (2023). Impact of severe polyhandicap on parents’ quality of life: a large French cross-sectional study. PLoS One 14:e0211640. doi: 10.1371/journal.pone.0211640, 30716093 PMC6361449

[ref80] Rubin H. J. Rubin I. (2012). Qualitative Interviewing: The Art of Hearing Data. 3rd Edn Thousand Oaks: Sage Publications.

[ref81] Schuelka M. J. (2013). A faith in humanness: disability, religion and development. Disabil. Soc. 28, 500–513. doi: 10.1080/09687599.2012.717880

[ref82] Shakespeare T. (1994). Cultural representation of disabled people: dustbins for disavowal? Disabil. Soc. 9, 283–299. doi: 10.1080/09687599466780341

[ref9001] Shakespeare T. (2006). Disability rights and wrongs. London: Routledge.

[ref83] Shapiro J. Monzó L. D. Rueda R. Gomez J. A. Blacher J. (2004). Alienated advocacy: perspectives of Latina mothers of young adults with developmental disabilities on service systems. Ment. Retard. 42, 37–54. doi: 10.1352/0047-6765(2004)42<37:AAPOLM>2.0.CO;2 14720095

[ref84] Singh N. N. Curtis W. J. Ellis C. R. Nicholson M. W. Villani T. M. Wechsler H. A. (1995). Psychometric analysis of the family empowerment scale. J. Emot. Behav. Disord. 3, 85–91. doi: 10.1177/106342669500300203

[ref85] Smith J. Cheater F. Bekker H. (2015). Parents’ experiences of living with a child with a long-term condition: a rapid structured review of the literature. Health Expect. 18, 452–474. doi: 10.1111/hex.12040 23311692 PMC5060798

[ref86] Sofokleous R. Stylianou S. (2023). Effects of exposure to medical model and social model online constructions of disability on attitudes toward wheelchair users: results from an online experiment. J. Creat. Commun. 18, 61–78. doi: 10.1177/09732586221136260

[ref87] Stoykova M. (2021). Empowerment and social functioning of people with mental disabilities. J. Reattach Therapy Dev. Diversit. 4, 39–49. doi: 10.26407/jrtdd2021.1.43

[ref88] Sulaimani G. H. Kamel S. Alotaibi G. Telmesani N. (2023). Quality of life among family caregivers of disabled children in Saudi Arabia. Cureus. 15:e41320. doi: 10.7759/cureus.41320, 37539394 PMC10395756

[ref89] Swain J. French S. (2000). Towards an affirmation model of disability. Disabil. Soc. 15, 569–582. doi: 10.1080/09687590050058189

[ref90] Szlamka Z. Tekola B. Hoekstra R. Hanlon C. (2022). The role of advocacy and empowerment in shaping service development for families raising children with developmental disabilities. Health Expect. 25, 1882–1891. doi: 10.1111/hex.13539, 35644908 PMC9327816

[ref91] Tadema A. C. Vlaskamp C. (2010). The time and effort in taking care for children with profound intellectual and multiple disabilities: a study on care load and support. Br. J. Learn. Disabil. 38, 41–48. doi: 10.1111/j.1468-3156.2009.00561.x

[ref92] Tenny S. Brannan J. M. Brannan G. D. (2022). Qualitative Study. Treasure Island: StatPearls Publishing. 29262162

[ref93] Tören Z. Aslan Açan B. (2024). Social workers’ experience with the concept of empowerment: voices from the disability field. J. Soc. Work. Pract. 38, 287–301. doi: 10.1080/02650533.2024.2379449

[ref94] Trainor A. A. (2010). Diverse approaches to parent advocacy during special education home–school interactions. Remedial Spec. Educ. 31, 34–47. doi: 10.1177/0741932508324401

[ref95] Wakimizu R. Fujioka H. Yoneyama A. Iejima A. Miyamoto S. (2011). Factors associated with the empowerment of Japanese families raising a child with developmental disorders. Res. Dev. Disabil. 32, 1030–1037. doi: 10.1016/j.ridd.2011.01.037, 21353460

[ref96] Wakimizu R. Matsuzawa A. Fujioka H. Nishigaki K. Sato I. Suzuki S. . (2022). Effectiveness of a peer group-based online intervention program in empowering families of children with disabilities at home. Front. Pediatr. 10:897584. doi: 10.3389/fped.2022.929146, 36353259 PMC9638189

[ref97] Wakimizu R. Yamaguchi K. Fujioka H. (2017). Family empowerment and quality of life of parents raising children with developmental disabilities in 78 Japanese families. Int. J. Nurs. Sci. 4, 38–45. doi: 10.1016/j.ijnss.2016.12.004, 31406716 PMC6626077

[ref98] Wakimizu R. Yamaguchi K. Fujioka H. Numaguchi C. Nishigaki K. Sato N. . (2016). Assessment of quality of life, family function, and family empowerment for families who provide home care for a child with severe motor and intellectual disabilities in Japan. Health 8, 304–317. doi: 10.4236/health.2016.84032

[ref99] Weiss J. A. Cappadocia M. C. MacMullin J. A. Viecili M. Lunsky Y. (2012). The impact of problem behaviours of children with ASD on parent mental health: the mediating role of acceptance and empowerment. Autism 16, 261–274. doi: 10.1177/1362361311422708, 22297202

[ref100] Winance M. (2007). Being normally different? Changes to normalisation processes: from alignment to work on the norm. Disabil. Soc. 22, 625–638. doi: 10.1080/09687590701560261

[ref101] Woodgate R. L. Edwards M. Ripat J. D. Borton B. Rempel G. (2015). Intense parenting: a qualitative study detailing the experiences of parenting children with complex care needs. J. Pediatr. Nurs. 15, 875–887. doi: 10.1186/s12887-015-0514-5 PMC4660805 26611116

[ref102] Zaks Z. (2023). Changing the medical model of disability to the normalisation model of disability: clarifying the past to create a new future direction. Disabil. Soc. 39, 3233–3260. doi: 10.1080/09687599.2023.2255926

